# Heating effect on quality characteristics of mixed canola cooking oils

**DOI:** 10.1186/s13065-022-00796-z

**Published:** 2022-01-17

**Authors:** Ayesha Baig, Muhammad Zubair, Sajjad Hussain Sumrra, Muhammad Faizan Nazar, Muhammad Nadeem Zafar, Kausar Jabeen, Muhammad Bilal Hassan, Umer Rashid

**Affiliations:** 1 Department of Chemistry, University of Gujrat, Gujrat, 50700 Pakistan; 2 Department of Chemistry, University of Education Lahore, Multan Campus, Lahore, Pakistan; 3 Department of Statistics, University of Gujrat, Gujrat, 50700 Pakistan; 4 Institute of Nanoscience and Nanotechnology (ION2), Universiti Putra Malaysia (UPM), 43400 Serdang, Selangor Malaysia

**Keywords:** Canola oils, Unsaturated, Fatty acids, Physicochemical, Un-saponifiable

## Abstract

**Background:**

The subcontinent is famous for its variety of seasonal foods cooked in vegetable seed cooking oils at elevated heating. Oils are often of poor quality that effect to consumer health. The work, therefore, planned to examine the effects of heat on the quality of mixed canola cooking oils (MCCOs). MCCOs were analyzed by preparing volatile fatty acid methyl esters (FAMEs) and for physiochemical properties.

**Results:**

A major change was observed in the FAs composition of various MCCOs as coded K-1 to K-5. MCCOs were found rich in unsaturated 9-octadecanoic acid (oleic acid C_18:1_) and 9, 12-octadecadienoic acid (linoleic acid C_18:2_) along saturated octadecanoic acid (stearic acid C_18:0_). Results reveals that canola oil samples are mixed in the range of 4–30% with other vegetable oils and animal fats. The quality of canola cooking oils further reduced after heating to 100 °C, 200 °C and 350 °C, respectively. Quality parameters of MCCOs were significantly altered after heating and found as color (510–520 nm to 570–600 nm), mass 220–237 g to 210–225 g, volume 250 mL to 239 mL, pH (6.76–6.89), specific gravity (0.87–0.92), refractive index (1.471–1.475), saponification value (SV) (0.7–2.5), un-saponifiable matter (2.4–9.8%) and acid value (AV) (1.20–5.0 mg KOH).

**Conclusion:**

Heating of oils at elevated temperature have shown a significant effect on pH, specific gravity and un-saponifiable matter (p-value < 0.05). Large changes in the physicochemical parameters and FAs composition help to develop a conclusion that cooking at high temperatures affects the quality of mixed canola cooking oils.

**Supplementary Information:**

The online version contains supplementary material available at 10.1186/s13065-022-00796-z.

## Introduction

Edible oils (EOs) contain high-energy dietary unsaturated FAs that are not only essential part of human nutrition but also contribute significantly in transportation of nutrients in the body [1]. Oil extracted from the vegetable seeds of different varieties used widely in communal foods as a nutrient and bioactive component in some medicines [2]. In general, these oils contain 98% triglycerides, 2% phospholipids, and a variety of hydrocarbons [3]. In most cases, unsaturated FAs in vegetable oils are higher than polyunsaturated FAs, which makes them healthier for body [4, 5]. Edible oil FAs are not only recognized as essential energy resource but also contribute as major building blocks in the hormones development in the body to control the systems [6]. FAs vary in the number of carbon atoms as well as in the position and geometry of the double bonds within the carbon chain. The availability of healthy food is a real need of the hour and thus it is now a hot issue of concern in the scientific community and the public media [7]. Frying is the most common process of cooking as it reflects the desired taste and quality of the food. Repeated use and heating of oil produces unwanted ingredients which adversely affects the quality of food and human health. When vegetable oil is heated for a long time in the open air at high temperature, various chemical reactions (hydrolysis, oxidation and polymerization) occurs. As a result of reactions, cooking oil produce volatile components which result into degradation the oil [8]. Vegetable oil is used for frying purpose almost in all the countries of the world, but heating standards are different everywhere. Upon heating, the FAs of vegetable oils are converted to carbonyl and peroxide as degraded products which reduces the oil’s nutrients, and can cause colon cancer, atherosclerosis and heart problems in humans [5]. Deep frying instigate chemical reactions that depend on the frying conditions, oil quality, oil conversion and oxidative stability. At higher temperatures, excess oxygen in the air becomes the reason to oxidize the oil, causing structural changes, which reduces the quality of the oil and makes it dangerous to human health [9]. The use of degraded oils for cooking at high temperatures has the following effects in humans: suppression of weight and growth, increase in liver and kidney, damage in thymus, and epididymis [8]. Research reveals that heating the vegetable oils up to the level of 180 °C changes their chemical composition and reduces the quality of oil by decomposing the heat-labile vitamin E [10]. Thermal oxidation and auto-oxidation are well-defined reaction in heated cooking oil that affect the stability of cooking oil. From a nutritional point of view, cooking oil should exhibit a high degree of oxidative stability. Vegetable oils possessing high levels of saturated FAs, including trans FAs, may become harmful for health [11]. Monitoring the quality of cooking oils is an essential obligation to avoid the harm of oil on health and to reduce the effects of food prepared in such oils and cost of disposing of cooking oils [4]. Oils having more unsaturation are more easily oxidized, such as 75–90% oleic acid oils are more resistant to heat degradation than 65% linoleic acid [12]. Both conventional flame and microwave heating augments the oxidation of fatty acids in cooking oil and rapidly reduces the unsaturated FAs. Over the last 20 years, Pakistan's production of edible oil from all sources has increased by 2.56% annually while consumption has increased by 7.7%, due to which local edible oil production could not meet the demand. Pakistan imports 75% of its total crude oil and as a result, local cooking oil production has been largely increased [13]. In Pakistan, tradition crops like rapeseed, mustard, groundnut, sesame and cotton, and non-traditional crops such as sunflower, soybean and safflower are grown to meet cooking oil needs. The Pakistan Agriculture Research Council (PARC) contributes to the growing deficit of canola based vegetable cooking oils (CBVCOs) in Pakistan through research on canola varieties. Now, the canola seed oil is a major contributor to the production of domestic CBVCOs. PARC for the first time extracted canola oil and marketed it as Mumta pure canola cooking oil, which resulted in the establishment of various industries in the country, selling canola cooking oil under different brand names. Therefore, in the national interest and to reduce foreign exchange, there is a need to increase domestic oil production. In addition, regulating hundreds of unregistered oil extraction units is a difficult task and any attempt to do so could fail, and many of jobs could be stopped altogether, leading to unemployment and oil shortages. Pakistan's Punjab province is known for its variety of food and beverages that produce unique flavors and aromas at high temperatures in vegetable oils. The oil is mainly heated to fry and roast a variety of foods, including vegetables, meat, grains, lentils and pulses. Edible vegetable oil is used 3–6 times before disposal. During the frying and cooking process, heating damages the oil and therefore reduces the quality of the food. The low quality of the oil may be due to poor industrial processing, mixing or adulteration of used cooking oil [14]. Locally processed cooking oils due to poor regulatory system provide insufficient information of cooking oil’s quality characteristics. In particular, cooking oils used for food preparation are hardened at high temperatures during frying, which can alter quality properties and generally make them unsafe for health and reuse. Therefore, the present study is designed with the main objective of heating effect on quality characteristics of mixed canola cooking oils.

## Materials and methods

### Sample collection, preservation and chemicals

In the present research work, five different commercially available MCCOs, from the market were purchased, preserved and used in research work. Canola cooking oils were labeled as Kisan (K-1), Kausar (K-2), Talu (K-3), Apna (K-4) and Sufi (K-5). All the oils were observed for their expiry date and shelf life to ensure basic food quality. The purchased oil was brought to the Research Laboratory of the Department of Chemistry, University of Gujarat, Pakistan and then stored in the refrigerator at 4 °C for later analysis. All of the chemicals (absolute ethanol, methanol, and glacial acetic acid) used in the research work were of high purity analytical grade, and they were purchased from Sigma Aldrich. Potassium hydroxide, sodium hydroxide, hydrochloric acid and sulfuric acid were from Lab Scan and Iso-octane, n-hexane and phenolphthalein were from Merck.

### Research design for the evaluation of MCCOs quality

For each heating phase, four equal portions of every oil 250 mL were taken, and heated and then cooled to normal temperature by covering with covered with aluminum foil and kept in the refrigerator. The physicochemical properties of the oil were tested in triplicate at control level and after the heating. Color on a spectrophotometer and the average maximum absorption wavelength (ʎ) was determined following the method of [15]. Mass and volume were calculated by following the specific gravity AOAC method, Official Method 920.212, pH by potentiometric (AOAC, Official Method 981.12), RI by digital refractometer (AOAC, Official Method 921.08), specific gravity by the pycnometer method (AOAC, Official Method 920.212), SV (AOAC, Official Method 920.160), AV (IUPAC Method No 2.201), un-saponifiable matter by extraction with petroleum ether (AOAC, Official Method 933.08), and water content was determined using standard protocols and methods. After the preliminary assessments, the oils were heated to 100 °C, 200 °C and 350 °C for 8 h according to traditional methods of frying. The oils were heated in a stainless steel shallow pan (20–30 cm in diameter) over a gas flame without adding any food to find the heating effect on the oil.

### Preparation and analysis of fatty acid methyl esters (FAMEs) of MCCOs

Fatty acids of all oils samples labeled as K-1, K-2, K-3, K-4 and K-5 were converted to methyl ester derivatives. An amount of 30 mg of oil was treated with 5.0 mL of 0.5 M methanolic KOH. The whole mixture was vortexed repeatedly and heated for two hours at 80 °C to obtain a monophasic layer. The samples were cooled to room temperature and then 0.3 mL each of glacial acetic acid, hexane, and distilled water were added until equilibrium was reached and the phase-separated. The upper layer containing FAMEs was transferred to a labelled sample GC vial using a pasture pipette. Prepared FAMEs sample was injected (splitless injection) into GC–MS (Shimadzu GC–MS Q 2010, SHIMADZU Corporation, Japan) to analyze the fatty acid composition. The GC–MS was fitted with a polar capillary column (Rtx-5MS, film thickness − 0.25 µm and length-30 m × 0.32 mm ID). The oven temperature was programmed to start from 160 °C for 1 min to 250 °C with a ramp rate of 5 °C/min and the last hold-up was 10 min. Helium (He) gas, 99.9% pure, was used as the carrier, with a flow rate of 1.0 mL/min throughout the analysis. The temperature of the interface, ion source and injection were 220 °C, 230 °C and 250 °C, respectively. The mass detector was operated in electron impact (EI) mode with an ionization energy of 70 eV and a m/z scan range of 30–500. Compound detection was made using GC–MS’s built-in NIST 2017 updated library. Triplicate injections were given and the method was > 90% accurate and precise with < 4% relative standard deviation (SD).

### Statistical analysis

Regression analysis was used to predict the desired values using the IBM SPSS statistics application. A one-way analysis of variability (ANOVA) was estimated to determine the significant difference (5%) between the groups of MCCOs (K-1 to K-5). The Tukey HSD test was then applied to groups where a significant difference was observed. Data for normality distribution was also tested by applying Shapiro–Wilk at the level of 0.05. Each experiment was performed in a triplicate manner and the results are reported as average ± SD.

## Results and discussion

### FA compositions of the MCCOs

**Fig. 1:**
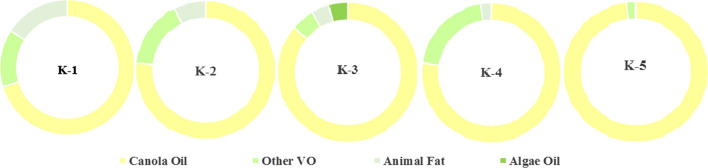
Different MCCOs comprising variations of different % composition

The literature reports that 9-octadecenoic acid (oleic acid C_18:1_) with linoleic (C_18:2_) and linolenic acids (C_18:3_) [16] as the main fatty acids in canola oils. Our results also show 9-octadecenoic acid (oleic acid C_18:1_) in the range of 404–572 mg/g, which represents a large proportion of canola FA (Table 1). In addition to the composition of important FAs, some oils contain hexadecanoic acid (C_16:0_) eicosanoic acid (C_20:0_), docosanoic acid (C_22:0_) and their derivatives 13-eicosanoic acid and 13-docosanoic acid in K-3, K-4 and K-5 respectively, and these findings were in agreement with those reported by Matthaus et al. [17]. Overall, GC–MS analysis reveals that MCCOs procured from the market contain a large proportion of canola oil, while some mixing of other vegetable oils, algae and animal is being made (Fig. 1). Oil K-1 contains about 70% canola FAs and 30% other sources such as animal fat 13% and edible oil 17%, K-2 76.5% canola FAs, 16% other sourced oil and 7.5% animal fat composition, K-3 86% canola oil 5.5% other vegetable oils, 4.4% algae and 4% animal fat, K-4 77.2% canola oil, 20.3% other source oil and 2.5% animal fat whereas K-5 contains 97.9% canola oil, 2.1% other source oil respectively. On comparing the quality prospective, K-5 found the best among the MCCOs because it contains about 98% canola oil and 2% other vegetable oils. Overall, the analysis reveals that the principle FAs of MCCOs are unsaturated FAs of oleic C_18:1_ (9-Octadecenoic acid 700–980 mg/g), linoleic C_18:2_ (9,12-Octadecenoic acid 160–230 mg/g), linolenic C_18:3_ (9,12,15-Octadecenoic acid 0–120 mg/g) and erucic acid C_22:1_ (13-eicosanoic acid 0–111 mg/g), and the saturated FAs of palmitic C_16:0_ (hexadecanoic acid 48–57 mg/g) and animal fat (16–85 mg/g). MCCOs showed slightly lower erucic and linolenic acid, but higher content of oleic acids and linoleic. Major FAs as detected in the canola oils were found to be similar to another study so there exit no significant difference [18]. The content of the FAs showed a notable variation in samples. The nutritional value of canola oil like other fats and oils, depends on the composition of their FAs, especially the amount of oleic, linoleic, linolenic, and erucic acids that are of great importance in human nutrition. In addition, high oleic acid oils have cholesterol-lowering properties. While saturated (palmitic and stearic) FAs significantly increase blood cholesterol levels [19]. At the same time, the dietary quality of the cooking vegetable seed oils is improved by increasing the content of dietary essential 9,12-octadecadienoic acid (C_18:2_) and reducing the content of 9,12,15-octadecadienoic acid (C_18:3_). Thus, edible oil with high linoleic acid content is a premium oil. On account of the results of GC–MS analysis, linoleic acid an essential FA found higher in all vegetable oils, especially in K-3, and its presence in the oil can be the major source of rancidity in vegetable oils. Research results has also depicts that MCCOs have mixing of other vegetable oils and animal fats in the form of octadecanoic acid and cyclopentadecanoic. MCCOs have been found to have 4–30% blending and had a relatively high oxidative stability and tasted better than other frying oils. The results show that mixed oil is a good alternative oil for frying foods [20].

**Table 1:** Fatty acids (mg/g of oil) of MCCOs (K-1 to K-5) analyzed by GC–MS

Fatty acid	FA groups	Source	K-1	K-2	K-3	K-4	K-5
9-Octadecenoic acid	MUFA	Canola	500	404	453	476	572
Hexadecanoic acid	SFA	Canola	–	127	52	57	48
9,12-octadecadienoic acid	MUFA	Canola	160	234	–	–	201
Eicosanoic acid	SFA	Canola	–	–	51	105	54
7-Hexadecenoic acid	MUFA	Other VO	–	–	42	06	21
Pentadecanoic acid	SFA	Animal fat	49	75	–	25	–
13-Docosanoic acid	MUFA	Canola	–	–	31	25	–
Docosanoic acid	SFA	Canola	–	–	41	–	22
9-Hexadecenoic acid	MUFA	Other VO	–	121	13	–	–
Cyclopropane octanoic acid	SFA	Animal Fat	87	–	16	–	–
Cyclo pentane tri decanoic acid	SFA	Other VO	–	39	–	20	–
13-Eicosanoic acid	MUFA	Canola	–	–	113	–	–
Oxiraneoctanoic acid	SFA	Other VO	67	–	–	89	–
6-Octadecanoic acid	MUFA	Canola	–	–	–	56	–
10-Octadecanoic acid	MUFA	Canola	–	–	–	54	–
Cyclo propanepentanoic acid	SFA	Other VO	–	–	–	34	–
9,12,15-Octadecatrienoic acid	PUFA	Canola	–	–	120	–	–
8,11-Octadecadenoic acid	PUFA	Algae	–	–	44	–	–
7-Hexadecenoic acid	MUFA	Other VO	98	–	–	–	–
Heneicosanoic acid	SFA	Other VO	–	–	–	22	–
Cyclo pentane-undecanoic acid	SFA	Other VO	–	–	–	18	–
Cyclo propanetridecanoic acid	SFA	Other VO	–	–	–	13	–
10-Octadecenoic acid	MUFA	Canola	–	–	–	–	82
7,10-Hexadecenoic acid	PUFA	Canola	39	–	–	–	–
Octadecanoic acid	SFA	Animal Fat	24	–	–	–	–

### Effect of heating on FAs of MCCOs

**Fig. 2:**
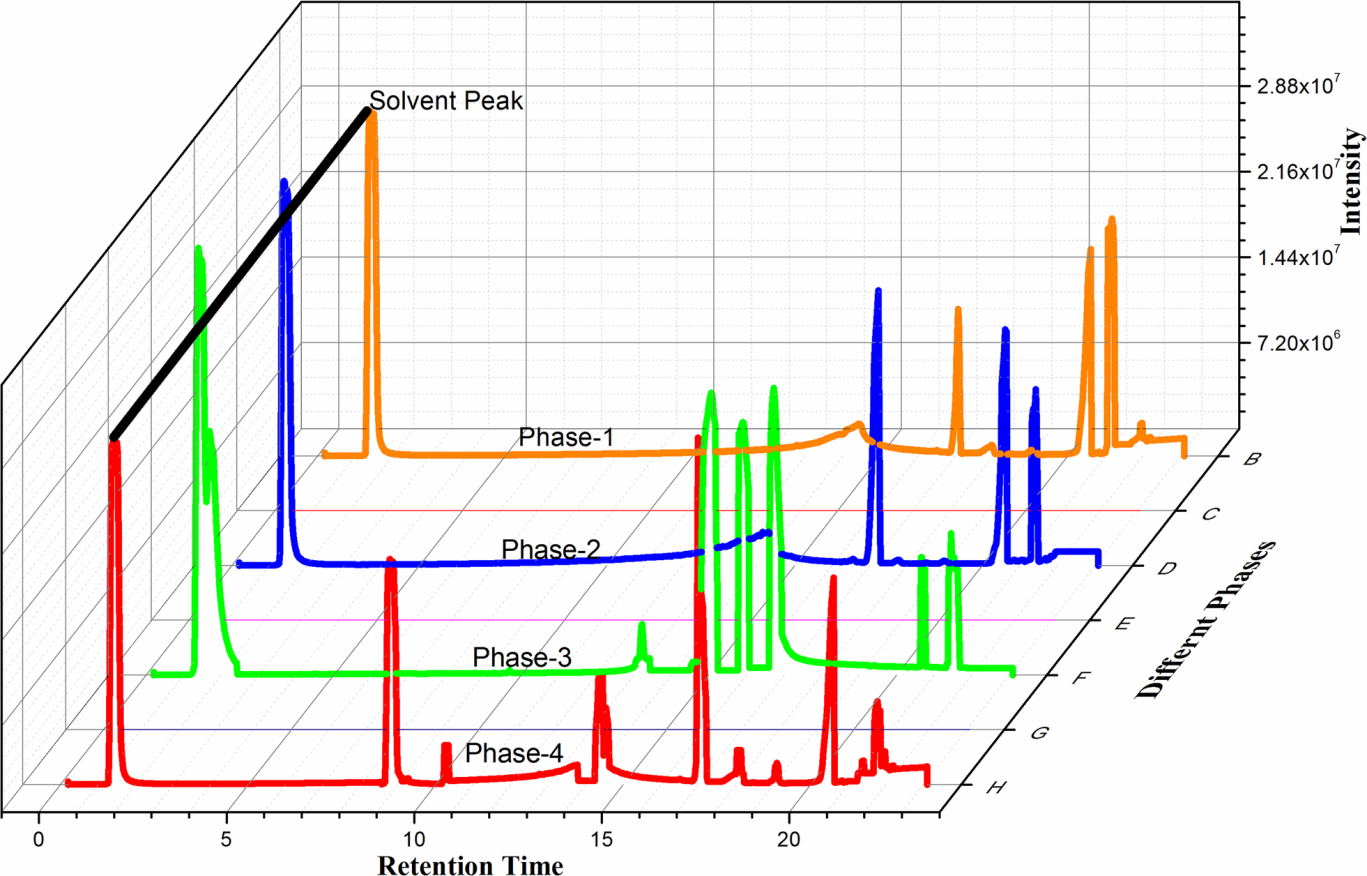
Representative GC–MS analysis depicts the effect of heating on the FAs of K-2 oil on different temperatures

The results show that free FAs increase with increasing temperature, which affects the bonding of triglycerides to thermal decomposition. It has been observed that at normal temperatures, the level of free FAs in vegetable oil is not very high and only a few FFAs have been detected in GS-MS analysis (Fig. 2) which supports the previous studies [21, 22]. Upon heating the oil to 100 °C, the level of FFAs begins to increase in thermal degradation. In the third and fourth stages, in addition to the initial stage, the FFA increased. With an increase in temperature from 25 to 200 °C, the level of polyunsaturated FAs (PUFAs) such as linoleic acid decreases (47 to 6.13 g/100 g of oil). This decrease in linoleic acid content during prolonged heating periods is due to an increase in its auto-oxidation rate with an increase in heating period [23]. The composition of free FAs varies significantly with increasing temperature from 200 to 350 °C. Similarly, in a research study by Dawodu et al., FFA increased by 2–3% in which the temperature rises to 250 °C which strongly supports the research carried out in the present study [24]. High levels of FFA in cooking oil are not suitable for human consumption because activity of the free fatty acids is postulated to be due to their ability to attract prooxidant.

### Effect of heating on color, mass and volume of MCCOs

Visual indication and fast way to test the quality of color oil. In this study, there is a significant change from the yellow color of the oil to the deep reddish color during heating (Table 2). As the heating temperature rises, the wavelength of the oil increases. Visually, the color of the oil first appears yellow (sample wavelength range 510–520 nm), then on continuous heating it turns to reddish brown (sample wavelength range 580–600 nm). Thus, it has been observed that darkening of oil color may be due to auto and thermal oxidation of phospholipids [25]. As the temperature rises, the formation of ketones, dienoic acids and peroxides increases, resulting in darkening of the oil [26]. Severe elevated heating affects the color of canola oils and other quality properties of vegetable oils. The literature states that aldehydes are formed during the heating of vegetable cooking oils such as coconut, safflower, canola and extra virgin olives. The oils were heated to 180 °C, 210 °C and 240 °C after 6 h and the emission is characterized by gas chromatography [27]. Cooking oils emit a mixture of chemicals on heating, from that the aldehydes have attracted great attention because many of them are hazardous, containing long-chains related to the FAs in cooking oils [28]. In present research, each type of oil emitted aroma upon heating with a strong intensity of smell which may have represented the presence of aldehydes. Evolution of oil aroma is also acceptable because of the change in the mass ad volume of the oils [29]. Literature reveals, that cooking oils at high temperatures enhance sensory functions, including the brown colour and unique fragrance. Chemical reactions alter the physicochemical properties of cooking oils and ultimately reduce their mass and volume, leading to the production of many by-products [30].

**Table 2:** Effect of Heating on Color, Mass and Volume of MCCOs

CBVCOs	Property	25 °C	100 °C/8 h	200 °C/8 h	350 °C/8 h
K-1	Color (ʎnm)	515	525	550	595
	Mass (g)	222.5	222.06	215.00	212.50
	Volume (mL)	250	246	244	242
K-2	Color (ʎnm)	510	520	555	590
	Mass (g)	220.00	219.57	215.00	210.00
	Volume (mL)	250	248	246	242
K-3	Color (ʎnm)	515	520	545	570
	Mass (g)	237.50	236.99	227.50	225.00
	Volume (mL)	250	243	241	239
K-4	Color (ʎnm)	520	530	560	600
	Mass (g)	225.00	224.64	215.00	212.50
	Volume (mL)	250	248	241	240
K-5	Color (ʎnm)	515	520	545	570
	Mass (g)	230.00	229.54	222.50	217.50
	Volume (mL)	250	246	243	239

### Effect of heating on the SV of the MCCOs

**Fig. 3:**
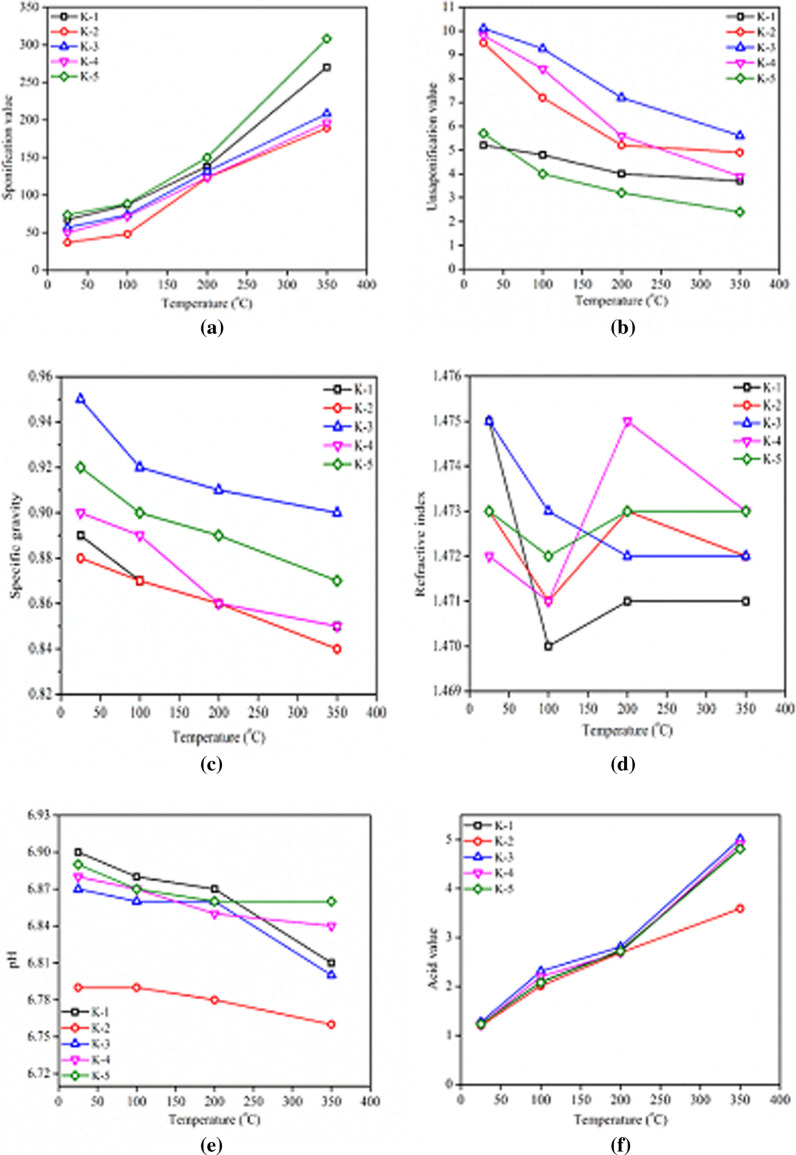
The effect of heating on quality parameters **a** saponification value, **b** un-saponifiable matter, **c** specific gravity **d** refractive index, **e** pH, **f** acid value of MCCOs

Saponification value (SV) provides very useful information and represent the optimized quality of cooking oil without the adulteration in the refined oils. The results are shown in Fig. 3a, which depicts the lower SV to be around 67 mg of KOH/100 g of an oil at room temperature. SV increased up to 250 mg of KOH/100 g of oil at the elevated cooking temperature of 350 °C. These findings are in close agreement with Odewole et al. [31]. The trend of increase in the SVs observed in all the selected MCCOs (K-1, K-5), which corresponds to increase in temperature. Highest increase in the SV was observed in the K-5 cooking oil in comparison to the other oils (Fig. 3a). Increasing trend reveals a significant change in the SV of the oils, which indicates that more free FAs may be producing during heating. This may be attributed to the transformation of the triglycerides to FFAs, which effect to increase the FFA content of the oils and, hence, enhanced the SVs [32]. According to the reported values in the literature, an optimized SV should be 182–193 mg of KOH/100 g of oil. The SV of many vegetable oils above the 200 mg/KOH indicates oil adulteration [33]. Table 3 represents the statistical description, giving the regression equations for the prediction of the SV by putting temperature as a factor of analysis. The one-way ANOVA was calculated based on the mean values rather than on the base values, depicting no significant variation in the SV (F_calculated_ < F_critical_) among the groups of the cooking oils treated at different temperatures.

**Table 3:** Statistical evaluation of effect of heating on different MCCOs

Characteristic	CBVCOs	Mean range	SD range	Df	Significant value	F-value	P-value	F_crit_
SV mg KOH	K-1–K-5	99.26–155.05	65.10–107.34	4	0.230–0.644	0.30	0.88	3.47
Un-sap Matter		3.82–8.03	0.694–2.67		0.426–0.817	3.59	0.05	3.48
Specific Gravity		0.87–0.92	0.017–0.024		0.488–0.995	6.77	0.007	3.47
Refractive Index		1.471–1.473	0.001–0.002		0.001–0.850	2.03	0.166	3.48
pH		6.78–6.87	014–039		0.071–0.714	5.83	0.011	3.48
AV mg KOH g^−1^		2.37–2.85	1.01–1.57		0.644–0.993	0.093	0.982	3.478

F > F_crit_ or P-value < 0.05: significant and F < F_crit_ or P-value > 0.05: non-significant

### Effect of heating on the un-saponifiable matter of the MCCOs

The un-saponifiable matter of vegetable cooking oil is a composition of very important naturally occurring antioxidants and vitamins that are decomposed at high temperatures. According to the codex standard, normally the value of an un-saponifiable matter in vegetable oil must be equal or less than 20%. Initially, at room temperature, the maximum un-saponifiable matter was observed in K-3 which was 10.10 g/100 g of oil; whereas, the lowest was in K-1, i.e., 5.20 g/100 g of the oil. After treatment at various high temperatures the amounts of the un-saponifiable matter were reduced to 5.60 g/100 g in K-3 and 2.40 g/100 g in K-5 (Fig. 3b). The results revealed a significant effect of heating on the amount of un-saponifiable matter that lowered the quality of the cooking oil. In (Table 3), the regression analysis for the un-saponifiable matter was calculated by putting temperature as a factor of analysis. The one-way ANOVA was calculated on the 5% confidence level and a significant difference between these groups (K-1, K-5) was found, therefore, the null hypothesis has been rejected. It may be stated that the mean values of the un-saponifiable matter were different. Un-saponifiable matter comprises various antioxidants, therefore, this part provides the stability to the cooking oil. Herchi and his coworkers also reported a steady decrease of un-saponifiable content upon the heating of flaxseed hull oil [34]. Similarly, Gertz et al. [35] observed that the refined oils were less stable at elevated temperatures as compared to non-refined oils, and there was a co-relationship between the oxidative stability and un-saponifiable matter of the vegetable oils. Tavakoli and coworkers observed that the major proportion of un-saponifiable matter was linked to the tocopherols, with demonstrating healthful effects as well as antioxidant activity. The content of the un-saponifiable matter in the vegetable cooking oils, usually ranged from 0.5 to 2.5% and the values in olive and *Pistacia khinjuk* fruit oil were 1.2% and 1.5%, respectively. Similarly, in addition to the prevention of diseases and biological activities in living organisms, polyphenols also provide desirable anti-oxidative properties in vegetable cooking oils [36]. In our assessment (Fig. 3b) of the cooking oils’ un-saponifiable matter values, an apparent decrease was observed up to 50% in all the mixed canola cooking oils.

### Effect of heating on the specific gravity of MCCOs

With an increase in the temperature of MCCOs from room temperature to 350 °C, a drastic effect of heating on lowering the specific gravity was observed. The effect is shown in Fig. 3c, with a major change from 0.95 to 0.90 in K-3. These results of our research are in close agreement with the findings of Negash et al. [37], who reported the mean specific gravity for imported and locally made oils in the range of 0.807–0.823. The statistical description based on the regression calculation and ANOVA in one way at a 5% significance level revealed that there was a significant difference between these groups (K-1, K-5), and, hence, the null hypothesis has been rejected (Table 3). The prediction revealed that the decrease in the mean value of the specific gravity inferred poor refining of the MCCOs, showing deviation from the standards developed by the WHO/FAO [38]. The decrease in the specific gravity of the MCCOs was due to the breakdown of the oil components and a decrease in the density of the oils. Idun-Acquah and his coworkers investigated that the mean specific gravity of the frying oil decreased from 0.899 to 0.887 upon heating at 180 °C during the frying of food [39]. The initial values for the specific gravity of MCCOs are in agreement with the results of [40, 41] who reported the specific gravity of canola oils is 0.920, and [42] noticed, that the 0.918 specific gravity for groundnut and 0.939 for neem seed was safe to use.

### Effect of heating on the RI of MCCOs

The Pre and post-treated MCCOs were tested for their refractive index and changes were observed to be from 1.470 to 1.475 (Fig. 3d) which is in close agreement with Bello et al. [41] for T. occidentalis oil (1.460); In previous research reports of [22, 43, 44] the RI values of conventional oils as soybean (1.466–1.470) and palm kernel (1.449–1.451) and for corn oil (1.467), olive oil (1.465) and virgin oil (1.468). The refractive indices of the oils remained almost constant as the values were the indication of their degrees of saturation and had slightly increased (0.005) due to the changes in color. There was a slight deviation in the refractive indices that varies from 1.470 to 1.475 in the canola oils upon being heated at high temperatures. Conclusively, the ANOVA in one way was applied on the RI values of different oils and no significant difference in their refractive indices was found based on the null hypothesis (Table 3). Godswill et al. reported slight changes in the RI of palm oil from the initial 1.4653 to 1.4655, sunflower oils from 1.4722 to 1.4725 and sesame oil from 1.4722 to 1.4723 after repeated frying [45]. The RI ranging from 0.1470 to 0.1475 of the canola oils possibly contained unsaturation in the FA sequencing as our research got similar values [46]. In another similar study, the refractive indices of the canola cooking oils were slightly increased from 1.4713 to 1.4740 upon heating, which supported our results changing from 1.470 to 1.475 [47]. The values of the RI of Canarium oil were 1.47 and 1.45, extracted by both mechanical pressing and solvent extraction, respectively [48]. The RI values determined for peanut and corn oil were 1.46 and 1.47, respectively. These values, found in our research, are in good agreement with the values reported by [49]. An increase in oil darkening, turbidity and viscosity could presumably be responsible for an increase in the RI value of some vegetable oils by 0.0007 [50].

### Effect of heating on the pH of MCCOs

The pH of cooking quality vegetable oil is generally kept neutral and usually ranges from 6.9 to 6.7. As the temperature rises, the pH of the oil decreases. Because high temperatures convert vegetable oil triglycerides into FFA. Figure 3e indicates a clear decrease in pH after heating the MCCOs. Overall, the highest pH (6.9–6.8) was noted in K-1 and the lowest pH value (6.7) was in K-2. Presumably, the formation of FFAs on thermal treatment are important dynamics of vegetable oils that may be related to pH reduction. Presumably, the formation of FFAs on thermal treatment are important dynamics of vegetable oils that may be related to pH reduction. Upon comparing our results with other research studies, it was revealed that our results are in agreement with Yusuf et al. where the pH of the groundnut oil decreased from 6.97 to 6.89 upon increases in the heating temperature from 70 to 100 °C [51]. The thermal process begins the hydrolysis of oil triglycerides, changing them into FFAs and glycerol, which influences the pH-parameter. Fresh vegetable oils are weak basic liquids with pH values more than 7 which decline, upon heating, into an acidic form because of the hydrolysis of the triglycerides [52]. Table 3 is a statistical comparison of the calculated and Tabulated F value and F_calculated_ > F_critical_ (0.09 > 3.48); hence, this reveals that there was no significant difference between the tested groups of oils K-1, K-5.

### Effect of heating on the AV of MCCOs

As the temperature increased from 25 C to 350° C, the AVs of MCCOs increased (Fig. 3f). The results of our finding are in close agreement with the AVs of flaxseed oil, i.e., from 1.5 mg KOH g^−1^ of oil to 2.9 mg KOH g^–1^ of oil when heated from room temperature to 110 °C as reported by Herchi and his coworkers [34]. Alajtal et al. reported 4.49 and 5.05 (mg KOH/g) AVs for the other cooking oils before and after the frying process [53]. The results support our AVs research findings; however, both of the oils differed in their KOH requirements. Comparison of F_calculated_ > F_critical_ (0.09 > 3.48) was made using the null hypothesis under the one-way ANOVA. It was found that there was a significant difference between the AVs of the MCCOs (Table 3). AVs indicate the number of FFAs and the suitability of the oil for degradation and the level of oxidative deterioration. FAs are usually found in the form of triglycerides, but they disrupt into FFAs during processing at high temperatures. This may be due to an increase in the activity of hydrolytic and lipolysis, which decomposes the glycerides in the oil as the operating temperature increases. An increasing AV is a sign of oil deterioration, which, in turn, is caused by the degradation of the chemical bonds in oil at high temperatures. High FFAs in the diet should be avoided, as it can impair the liver's ability to store sugar [24] and can lead to heart disease. Bello et al. reported that 0.00 to 3.00 mg KOH/g of AVs of oil is required for the use of oil for food preparation. This suggests that vegetable cooking oil is safe for heating up to 200 °C [41].

### Statistical analysis

Standards of statistical analysis reveals that if significance value of Shapiro–Wilk is greater than 0.05, then it can be stated that the data is not significant and normally distributed (Table 3). It is evident and clearly reported in earlier statistical studies that non-significant results do not need further analysis particularly with tests like Tukey, Pearson, Duncan multiple, etc. The Tukey HSD (Honestly Significant Differences) test is used for finding the significant differences among groups. That’s why the Tukey HSD test is applied to only significant values in a preliminary ANOVA for study groups of un-saponifiable matter and a pH value having p < 0.05 (Additional file 1: Tables S1 and S2). So, the analysis revealed each pair in respect to its significance. If the observed significance values were less than 0.05, then it could be stated that there was a significant difference among the pairs. The analysis illustrates that groups as (K-1, K-5), (K-2, K-3), (K-3, K-2), (K-3, K-4), (K-4-K-2) (K-4-K-3) and (K-5-K-1) in pairs had no significant differences which cannot be observed by the simple ANOVA. Whereas, all other pairs had significant results. Having a non-significant difference meant that there is no considerable impact from the heating on that physiochemical parameter and vice versa. Similarly, for the pH value, when tested as an independent variable using the Tukey HSD test by putting a 0.05 level of significance, it was observed that the (K-1-K-3), (K-1-K-4), (K-1, K-5), (K-3, K-1), (K-3, K-4), (K-3, K-5), (K-4, K-1), (K-5-K-3), (K-4, K-5), (K-5, K-1), (K-5, K-3) and (K-5, K-4) pairs had no significant difference, which was not observed by the simple ANOVA. It is now clear to express that all the other pairs of the pH values had significant results.

## Conclusions

The study considered five MCCOs which are most commonly used at home kitchen and public restaurant for cooking the food across the country. To confirm the compositional changes in the oils, GC–MS analysis of all the MCCOs was performed in two dimensionally, on one way, it was found that these oils have large variations in FAs composition and on other way, they were found impure canola oils having mixing of other vegetable oil and animal fats. The analysis shows that MCCOs have mixing up to 30% with cooking oil to get the desired taste, which is a key factor in selling cooking oil. The quality parameters of MCCOs were assessed initially after purchase and later after heating at different temperatures. The results differ from the Codex Alimentarius Commission’s standards for rapeseed (canola) oil. Colour wavelength was changed (510–600 nm) which represents the darkness in colour. Similarly, mass, volume, un-saponifiable matter and pH were drastically changed while AV, RI, specific gravity less varied at high temperature as MCCOs heated. ANOVA predicted significant changes in the un-saponifiable matter and the pH of the oils. Based on the results of the research, it may be concluded that the canola oils which are being used in cooking are not of pure quality and heat affects their quality attributes. Canola oil should not be heated at higher temperatures to save changes in their quality parameters. Furthermore, it is also suggested that these should be oils used once for cooking and later may be converted into some value-added products to achieve economic sustainability.

## Supplementary Information

**Additional file 1:** **Table S1.** Multiple comparisons applying Tukey HSD test of sample groups for unsap. matter. **Table S2.** Multiple comparisons applying Tukey HSD test of sample groups for pH.

## Data Availability

The authors confirm that the data supporting the findings of this study are available within the article [and/or] its Additional file.
