# Supplementary material for: Heating effect on quality characteristics of mixed canola cooking oils
Source: BMC Chem. 2022 Jan 17;16(1):3. doi: 10.1186/s13065-022-00796-z (PMC8764801; doi:10.1186/s13065-022-00796-z)
Supplement: Supplementary file 1 — Additional file 1: Table S1. Multiple comparisons applying Tukey HSD test of sample groups for unsap. matter. Table S2. Multiple comparisons applying Tukey HSD test of sample groups for pH. [file 13065_2022_796_MOESM1_ESM.docx]

**Addiitonal file 1**

**Table S1.** Multiple comparisons applying Tukey HSD test of sample groups for unsap. matter

| I | J | Mean Difference  (I-J) | Standard  Error | Significance | 95% Confidence Interval | |
| --- | --- | --- | --- | --- | --- | --- |
|  |  |  |  |  | Lower Bound | Upper Bound |
| K1 | K2 | -2.27500 | 1.35113 | .472 | -6.4472 | 1.8972 |
|  | K3 | -3.61250 | 1.35113 | .106 | -7.7847 | .5597 |
|  | K4 | -2.50250 | 1.35113 | .382 | -6.6747 | 1.6697 |
|  | K5 | .60000 | 1.35113 | .991 | -3.5722 | 4.7722 |
| K2 | K1 | 2.27500 | 1.35113 | .472 | -1.8972 | 6.4472 |
|  | K3 | -1.33750 | 1.35113 | .856 | -5.5097 | 2.8347 |
|  | K4 | -.22750 | 1.35113 | 1.0 | -4.3997 | 3.9447 |
|  | K5 | 2.87500 | 1.35113 | .259 | -1.2972 | 7.0472 |
| K3 | K1 | 3.61250 | 1.35113 | .106 | -.5597 | 7.7847 |
|  | K2 | 1.33750 | 1.35113 | .856 | -2.8347 | 5.5097 |
|  | K4 | 1.11000 | 1.35113 | .920 | -3.0622 | 5.2822 |
|  | K5 | 4.21250^*^ | 1.35113 | .047 | .0403 | 8.3847 |
| K4 | K1 | 2.50250 | 1.35113 | .382 | -1.6697 | 6.6747 |
|  | K2 | .22750 | 1.35113 | 1.00 | -3.9447 | 4.3997 |
|  | K3 | -1.11000 | 1.35113 | .920 | -5.2822 | 3.0622 |
|  | K5 | 3.10250 | 1.35113 | .200 | -1.0697 | 7.2747 |
| K5 | K1 | -.60000 | 1.35113 | .991 | -4.7722 | 3.5722 |
|  | K2 | -2.87500 | 1.35113 | .259 | -7.0472 | 1.2972 |
|  | K3 | -4.21250^*^ | 1.35113 | .047 | -8.3847 | -.0403 |
|  | K4 | -3.10250 | 1.35113 | .200 | -7.2747 | 1.0697 |

*. The mean difference is significant at the 0.05 level

**Table S2.** Multiple comparisons applying Tukey HSD test of sample groups for pH

| I | J | Mean Difference  (I-J) | Standard  Error | Significance | 95% Confidence Interval | |
| --- | --- | --- | --- | --- | --- | --- |
|  |  |  |  |  | Lower Bound | Upper Bound |
| K-1 | K-2 | .08500^*^ | .01805 | .002 | .0293 | .1407 |
|  | K-3 | .01750 | .01805 | .865 | -.0382 | .0732 |
|  | K-4 | .00500 | .01805 | .999 | -.0507 | .0607 |
|  | K-5 | -.00500 | .01805 | .999 | -.0607 | .0507 |
| K-2 | K-1 | -.08500^*^ | .01805 | .002 | -.1407 | -.0293 |
|  | K-3 | -.06750^*^ | .01805 | .014 | -.1232 | -.0118 |
|  | K-4 | -.08000^*^ | .01805 | .004 | -.1357 | -.0243 |
|  | K-5 | -.09000^*^ | .01805 | .001 | -.1457 | -.0343 |
| K-3 | K-1 | -.01750 | .01805 | .865 | -.0732 | .0382 |
|  | K-2 | .06750^*^ | .01805 | .014 | .0118 | .1232 |
|  | K-4 | -.01250 | .01805 | .955 | -.0682 | .0432 |
|  | K-5 | -.02250 | .01805 | .726 | -.0782 | .0332 |
| K-4 | K-1 | -.00500 | .01805 | .999 | -.0607 | .0507 |
|  | K-2 | .08000^*^ | .01805 | .004 | .0243 | .1357 |
|  | K-3 | .01250 | .01805 | .955 | -.0432 | .0682 |
|  | K-5 | -.01000 | .01805 | .980 | -.0657 | .0457 |
| K-5 | K-1 | .00500 | .01805 | .999 | -.0507 | .0607 |
|  | K-2 | .09000^*^ | .01805 | .001 | .0343 | .1457 |
|  | K-3 | .02250 | .01805 | .726 | -.0332 | .0782 |
|  | K-4 | .01000 | .01805 | .980 | -.0457 | .0657 |

*. The mean difference is significant at the 0.05 level
